# The Use of Molecular Oxygen for Liquid Phase Aerobic Oxidations in Continuous Flow

**DOI:** 10.1007/s41061-018-0226-z

**Published:** 2018-12-11

**Authors:** Christopher A. Hone, C. Oliver Kappe

**Affiliations:** 1 Center for Continuous Synthesis and Processing (CCFLOW), Research Center Pharmaceutical Engineering (RCPE), Inffeldgasse 13, 8010 Graz, Austria; 2 Institute of Chemistry, NAWI Graz, University of Graz, Heinrichstrasse 28, 8010 Graz, Austria

**Keywords:** Continuous flow, Flow reactor, Continuous processing, Aerobic oxidation, Molecular oxygen, Process intensification, Membranes, Photochemistry, Green solvents

## Abstract

Molecular oxygen (O_2_) is the ultimate “green” oxidant for organic synthesis. There has been recent intensive research within the synthetic community to develop new selective liquid phase aerobic oxidation methodologies as a response to the necessity to reduce the environmental impact of chemical synthesis and manufacture. Green and sustainable chemical processes rely not only on effective chemistry but also on the implementation of reactor technologies that enhance reaction performance and overall safety. Continuous flow reactors have facilitated safer and more efficient utilization of O_2_, whilst enabling protocols to be scalable. In this article, we discuss recent advancements in the utilization of continuous processing for aerobic oxidations. The translation of aerobic oxidation from batch protocols to continuous flow processes, including process intensification (high T/p), is examined. The use of “synthetic air”, typically consisting of less than 10% O_2_ in N_2_, is compared to pure O_2_ (100% O_2_) as an oxidant source in terms of process efficiency and safety. Examples of homogeneous catalysis and heterogeneous (packed bed) catalysis are provided. The application of flow photoreactors for the in situ formation of singlet oxygen (^1^O_2_) for use in organic reactions, as well as the implementation of membrane technologies, green solvents and recent reactor solutions for handling O_2_ are covered.

## Introduction

Molecular oxygen (O_2_) is inexpensive, the most readily available oxidant on Earth, and completely harmless to the environment. O_2_ is therefore perhaps the greenest reagent available to the organic chemist [1]. Furthermore, O_2_ is a nontoxic gas and is easy to remove after a reaction. Aerobic oxidation reactions are generally very green because they typically display high atom economy and, in most cases, water is the only stoichiometric byproduct. Until very recently, classical oxidation methods using stoichiometric quantities of toxic inorganic oxidants, such as CrO_3_, KMnO_4_ and MnO_2_, were favored in organic synthesis, even though these protocols generally display poor atom economy and use highly energetic oxidants [2]. More recently adopted oxidation approaches use less toxic oxidants, such as dimethylsulfoxide (DMSO) and hypervalent iodine compounds, but are no less green. As social concern regarding the environmental impact of chemical processes gains more interest, there is an increasing demand to design more sustainable chemical methodologies. Anastas introduced the 12 principles of green chemistry, outlining the steps necessary for more sustainable synthesis practices [3]. Over the last 10–15 years, groundbreaking progress has been made in the development of highly selective aerobic oxidation reactions [4]. The replacement of toxic and corrosive stoichiometric oxidants with processes that use O_2_ combined with catalytic methodologies will ensure atom efficient and selective synthetic oxidation approaches that are sustainable into the future [5].

Oxidation chemistry utilizing pure O_2_ or air as the oxidant source is already used extensively within the bulk and commodity chemical manufacturing sector [6]. For example, 6 basic chemicals are produced using pure O_2_ and 12 chemicals using air at > 2 Mt/a scale. In the bulk and commodity chemicals sector, the use of air and O_2_ as the oxidant source is driven by the requirement to keep costs as low as possible. However, O_2_ is underutilized as an oxidant within the fine and pharmaceutical chemical industry. The bulk chemicals sector deals with low value, high volume products and the corresponding production plants are generally designed and engineered as dedicated continuous processes, whereas fine chemicals and the pharmaceutical sector have historically favored the use of multipurpose batch reactors for the manufacture of high value, low volume products [7]. There are unique process challenges associated with handling gas–liquid transformations within multipurpose batch reactors. Efficient mixing between the liquid phase and gas phase is difficult to achieve; therefore, reactions are often mass transfer limited, which leads to problems when scaling up from laboratory to manufacturing scale. The solubility of O_2_ in water and organic solvents is poor, thus the reactor needs to be pressurized to maximize the amount of gas in solution to reduce mass transfer effects. Typical commercial scale batch reactors can operate between 2 and 6 bar; therefore, higher pressures require more specialized and expensive equipment. In addition, aerobic oxidation reactions are typically highly exothermic, meaning the heat generated needs to be efficiently removed. These challenges, and the fact that the reaction utilizes potentially flammable O_2_ under certain conditions, unfortunately increase the perceived scale-up risk, which has rendered the use of O_2_ virtually unacceptable for pharmaceutical and fine chemical synthesis.

The challenges associated with handling O_2_ are better addressed by using continuous processing than multipurpose batch reactors [7, 8]. There is a current paradigm shift in the pharmaceutical industry from traditional batch manufacturing to continuous processing for the preparation of active pharmaceutical ingredients (APIs) [9–12]. This paradigm shift is reflected by a new focus in the pharmaceutical industry on process intensification, sustainability, product quality, safety, energy usage and cost [13]. The United States Food and Drug Administration (FDA) is taking proactive steps to facilitate the implementation of continuous manufacturing within the pharmaceutical industry as an attempt to improve product quality and reduce the environmental impact of pharmaceutical manufacture [14]. The University of Wisconsin-Madison Oxidation Consortium (MadOx) involving Eli Lilly and Co., Merck and Pfizer was established in 2012 as a precompetitive collaboration aimed at solving the challenges associated with aerobic oxidations in pharmaceutical manufacturing [15]. In particular, the consortium focused on the development of safe and scalable continuous flow technologies for aerobic oxidation reactions. Recent reviews have provided overviews of the significant progress made in the last decade towards the utilization of O_2_ within continuous flow environments [16–18].

A significant obstacle to the uptake of aerobic oxidation reactions is that undergraduate organic chemistry textbooks still teach classical oxidation methods, which use toxic inorganic oxidants in stoichiometric quantities rather than more recently developed greener aerobic oxidation strategies. Therefore, organic chemists lack the necessary knowledge to implement these new greener methods. There are hurdles to the implementation of large-scale aerobic oxidations owing to the lack of experience and equipment within pharmaceutical manufacturing. In this article, we highlight selected synthetic examples of liquid phase aerobic oxidation reactions under continuous flow conditions. The first section deals with the process aspects associated with utilizing aerobic oxidation reactions, and also gives an overview of a typical continuous flow setup for performing aerobic oxidations. Subsequently, homogeneous catalysis and heterogeneous catalysis examples are discussed. The utilization of photochemistry for the in situ formation of singlet oxygen (^1^O_2_) from ground state triplet oxygen (^3^O_2_) is treated only briefly, owing to the large number of examples published. The use of supercritical fluids and liquid carbon dioxide (CO_2_) as green solvents for aerobic oxidations is examined. Membrane technologies, new reactor developments and scale-up strategies are discussed. The advantages and challenges associated with the utilization of continuous processing for liquid phase aerobic oxidations are highlighted throughout.

## Process Aspects

### Mass and Heat Transfer

**Fig. 1:**
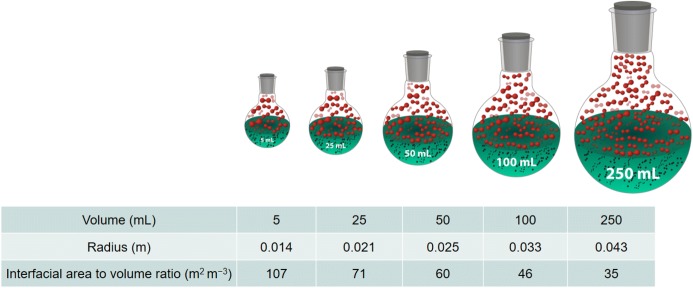
Interfacial area to volume ratio for laboratory batch reactors. Adapted from [21]

The solubility of O_2_ in organic solvents and water is generally very poor; therefore, the reaction rate for liquid phase aerobic oxidations in many cases is determined by mass transfer from the gas phase to the liquid phase [19]. The solubility of O_2_ in the liquid phase obeys Henry’s law whereby the amount of dissolved gas is proportional to its partial pressure in the gas phase [20]. Continuous flow reactors have advantages over standard glassware and sealed batch autoclaves in terms of mass transfer, even at a laboratory scale [21]. Within a batch processing environment, much of the gas is in the headspace, thus the reactor needs to be pressurized to maximize the amount of gas in solution. The rate of mass transfer from the gas phase to the liquid phase is also dependent on the interfacial contact area between the gas and liquid phases. The gas–liquid interfacial area to volume ratio decreases considerably with increasing batch reactor size [22] (see Fig. 1). Consequently, the results achieved within a small scale batch reactor are often irreproducible even within a laboratory batch reactor of slightly different dimensions. The highly exothermic nature of many aerobic oxidations also creates the need for efficient heat removal of the heat generated in the reaction to avoid thermal runaways. The reduction in reactor surface area to volume ratio with increasing reactor size makes it more difficult to remove the heat generated from a reaction at larger scales.

### Technology

**Fig. 2:**
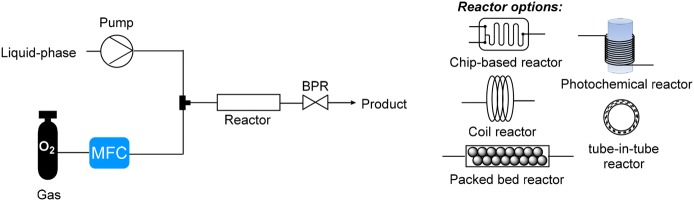
Simplified representation of a flow reactor configuration for liquid phase aerobic oxidation

**Fig. 3:**
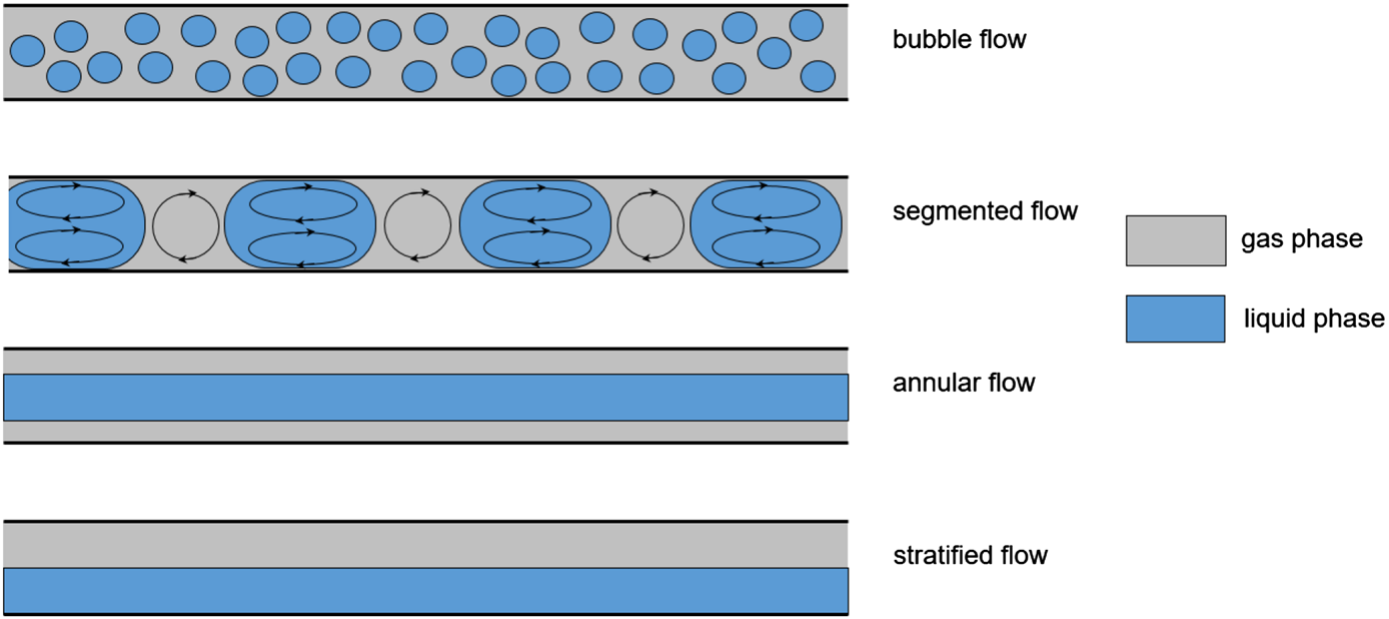
Flow regimes observed for gas–liquid mixtures within tubular reactors

A simplified representation of a gas–liquid flow system for liquid phase aerobic oxidations is shown in Fig. 2. A mass flow controller (MFC) is used to introduce O_2_ (or diluted O_2_) in a controlled manner directly from a cylinder. The liquid feed is usually introduced using a pump, either a HPLC, syringe or peristaltic pump. A typical reactor is either a chip-based, tubular coil, packed bed catalyst, photochemical or tube-in-tube system. A back pressure regulator (BPR) is used to control the system pressure. When the O_2_ gas is not fully dissolved within the liquid phase, different flow regimes can occur within a flow system (Fig. 3), with the exact flow regime depending on the gas and liquid flow rates, channel pattern and dimensions, and the physical properties of the fluid and gas composition. By far the most commonly observed flow regime within microchannels for liquid phase aerobic oxidations is a gas–liquid segmented (Taylor or slug) flow regime. The small vortices, known as toroidal currents, created inside each segment within a segmented flow regime result in enhanced mass transfer [23]. Typical interfacial area to volume ratio value ranges for different reactor types are shown in Table 1 [22]. The small channel dimensions of continuous flow reactors provide a high reactor surface area to volume ratio, enabling the generated heat to be dissipated quickly and allowing precise control of the reaction temperature.

**Table 1:** Interfacial area to volume ratio for different reactor types (data from [22])

Type of reactor	Interfacial area to volume ratio (m^2^ m^−3^)
Bubble columns	50–60
Impinging jet absorbers	90–2050
Packed columns, concurrent	10–1700
Packed columns, counter current	10–350
Static mixer	100–1000
Laboratory scale stirred tank (Fig. 1)	35–110
Stirred tank	100–2000
Tube reactors, horizontal and coil (Fig. 2)	50–700
Tube reactors, vertical	100–2000
Gas–liquid microchannel contactor	3400–18,000

### Using Diluted O_2_

The main challenge associated with the adoption of aerobic oxidation reactions in the pharmaceutical and fine chemical industry is the concern over safety due to the high risk of fires and explosions when flammable organic solvents and O_2_ are used in combination [24]. The combination of oxygen, organic solvent as a fuel and an ignition source (from a spark, flame, static electricity or heat) results in a potentially flammable mixture, because it satisfies the flammability triangle and thus the conditions for combustion to occur. A common strategy applied in batch manufacturing is to operate below the limiting oxygen concentration (LOC) value by diluting O_2_ gas with an inert gas, typically consisting of less than 10% O_2_ in N_2_ (“synthetic air”), to ensure the system never enters the explosive regime. The LOC value is defined as “the minimum partial pressure of oxygen that supports a combustible mixture”. Stahl and co-workers determined the LOC values experimentally in nine different solvents at elevated temperatures and pressures to ensure that a system could be safely operated without entering the explosive regime (Table 2) [25]. The benefit of operating at such low oxygen concentrations is that it ensures the process is inherently safe because a combustible mixture can never be formed.

**Table 2:** Limiting oxygen concentration (LOC) data for organic solvents.* NMP* *N*-Methyl-2-pyrrolidone,* DMSO* dimethylsulfoxide,* 2-MeTHF* 2-methyltetrahydrofuran (data from [25])

Solvent	Temperature (°C)	LOC (vol %)		
		1 bar	10 bar	20 bar
Acetic acid	200	10.6		9.6
NMP	200	8.1		7.6
DMSO	200	3.9		
DMSO	100	6.4		
*tert*-Amyl alcohol	100	9.6		10.1
Ethyl acetate	100	9.4		9.9
2-MeTHF	100	9.4		9.1
Methanol	100	7.6		6.9
Acetonitrile	100		12.1	11.9
Toluene	100	10.4	10.3	9.9
Toluene	25	11.6		
Methanol	25	8.6		
Acetone	25	12.7		

### Ability to Use Pure O_2_

**Fig. 4:**
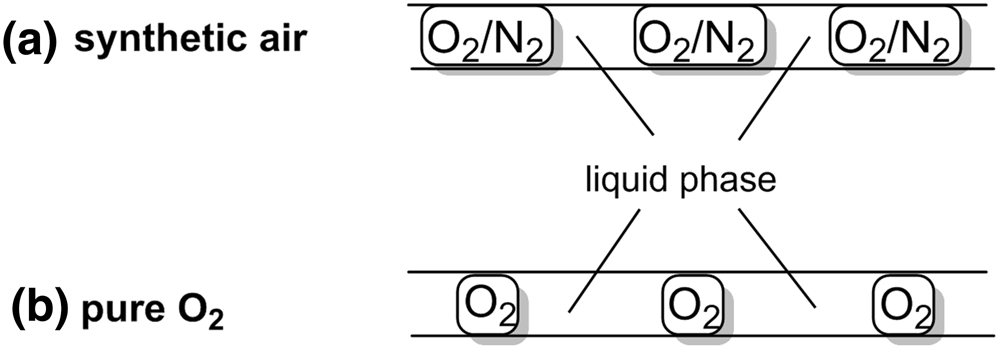
Illustrative example showing the gas contribution within a flow system for **a** synthetic air and **b** pure O_2_

Increasing reaction efficiency is fundamental to chemistry. The safe utilization of pure O_2_ at intensified conditions has been demonstrated on a number of liquid phase aerobic oxidation reactions. The limitation of using a diluted form of O_2_, for example 10% O_2_ in N_2_, is that the O_2_ is competing with N_2_ for dissolution in the liquid phase, therefore the reaction is more likely to be mass transfer limited (Fig. 4a) [18]. A substantially enhanced reaction rate can be achieved by using higher concentrations of O_2_, and even pure O_2_. The significant improvements in reaction rate achieved by using pure O_2_ can result in improved product quality and process efficiency. Superior space time yields (i.e., the product yield per unit of time and per reactor volume) can be achieved by using pure O_2_ compared to using synthetic air because a smaller gas phase is needed for the reactor (Fig. 4b). The utilization of pure O_2_ may also allow a lower system pressure to be used.

The minimum ignition energy (MIE) is the lowest energy required for an oxygen/organic vapor mixture to spontaneously ignite [26]. The MIE of flammable mixtures are over ten orders of magnitude lower for pure O_2_ than for air. Most safety studies carried out in microreactors examine the use of O_2_ for reactions occurring in the gas phase. Veser demonstrated for a Pt-catalyzed H_2_/O_2_ reaction to H_2_O_2_ that explosion propagation can be completely suppressed at channel sizes below the millimeter range, and thus the process is inherently safe [27]. However, at larger channel dimensions (> 0.4 cm) Poliakoff and co-workers, when investigating the catalytic dehydrogenation of 4-vinylcyclohexane, observed periodic temperature spikes near the surface of the Pd/Al_2_O_3_ catalyst bed that indicated the occurrence of cycles of propagating flames [28]. In the case of liquid phase aerobic oxidation reactions, O_2_ is substoichiometric to solvent, which significantly reduces the likelihood of an explosion. Small oxygen segments alleviate the likelihood that autoignition will occur, because the small channel dimensions do not exceed typical quenching distances for explosion propagation. Furthermore, the solvent plays a role as a heat sink. Unlike batch reactors, tubular flow reactors possess no headspace; therefore there is no headspace containing a large volume of potentially combustible oxygen/organic vapor. Nonetheless, the safety associated with a process should be assessed carefully on a case by case basis. Safe operation can be ensured by employing a properly designed continuous flow reactor that can withstand an explosion event in a worst case scenario [18]. A key benefit of continuous processing is that, generally, a far smaller inventory of the overall material to be processed is present within the system at any one time. Miniaturization reduces the risks and allows for secondary containment of the reactor in the case of an explosion event.

### Scale-up and Manufacture

When there is sufficient understanding of a reaction system and adequate process design to address safety concerns and mitigate risks, aerobic oxidations, even using pure O_2_, can be adopted at large scales through the utilization of appropriate continuous-flow processing systems. Experiments including microcalorimetry, differential scanning calorimetry (DSC) and autoclave explosion pressure measurements should focus on minimizing the perceived scale-up risk through contingency planning for worst case scenarios [29]. There are a number of scale-up strategies that can be applied, including: (1) running the process for a longer time in the same equipment (scale-out); (2) a larger reactor volume with the same channel diameter but faster flow rates; (3) unit parallelization (numbering up); and (4) channel dimension enlarging to provide a larger volume through smart dimensioning [30]. Examples of all of these strategies are shown below.

## Homogeneous Catalysis

### Pd-Catalyzed Reactions

Oxygen in its ground state, triplet oxygen (^3^O_2_), displays relatively low reactivity and poor selectivity; therefore, a catalyst system and/or elevated temperatures and pressures are required to increase reaction rates and improve selectivity. Palladium is perhaps the most studied metal for homogeneous catalyzed aerobic oxidations. A broad range of homogeneous Pd-catalyzed aerobic oxidations reactions have been developed over the last 10–15 years [31]. Palladium catalysts are very sensitive to the oxygen concentration. Pd(II) is reduced to Pd(0) species, which aggregate to form inactive Pd black [32]. This phenomenon causes a significant challenge when attempting to scale-up this chemistry under batch conditions due to poor mixing and temperature control. The direct oxidation of Pd(0) by O_2_ is kinetically unfavored. With this in mind, the utilization of continuous flow reactors that provide good heat and mass transfer can be beneficial for this type of chemistry by preventing catalyst decomposition through the rapid reoxidation of Pd(0) to Pd(II).

**Scheme 1:**
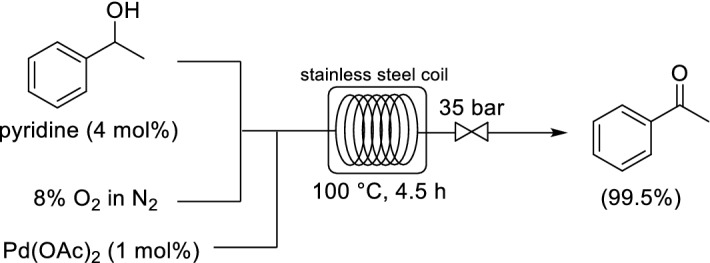
Continuous flow Pd-catalyzed aerobic oxidation of 1-phenylethanol to acetophenone

In collaboration with Eli Lilly and Co., Stahl and co-workers reported a continuous-flow setup for the Pd-catalyzed aerobic oxidation of alcohols to their corresponding aldehydes and ketones [33]. The system utilized a homogeneous Pd(OAc)_2_/pyridine catalyst system and a diluted oxygen gas source (8% O_2_ in N_2_). As stated in the Introduction, the main benefit of operating at such low oxygen concentrations is that it ensures that the oxygen/organic vapor will never enter the explosive regime, which makes the process inherently safe. A segmented (Taylor) flow regime provided a large interfacial area between the gas and liquid phases to increase mass transfer. The system was applied for the oxidation of primary and secondary alcohols (ten examples, 76–93% yields) within a 400 mL flow reactor. The oxidation of 1-phenylethanol to acetophenone was demonstrated within a 7 L stainless steel coil flow reactor at a 1 kg scale (Scheme 1). The limitation of Pd catalysts is the low catalytic turnover rate; therefore, in this case, a relatively long residence time (4.5 h) was necessary. Eli Lilly and Co. calculated that it is possible to scale-up from the 7 L reactor vessel by a minimum of two orders of magnitude, while retaining the high pressure rating, low cost, and a length/diameter ratio for the tube of ≥ 20,000/1.

**Scheme 2:**
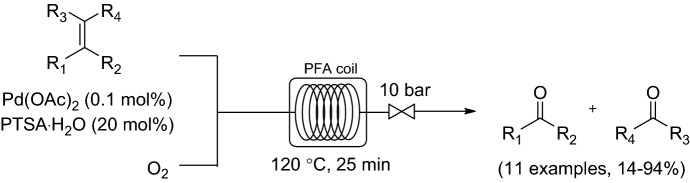
Continuous flow oxidative olefin cleavage to aldehydes and ketones

An instructive example for catalyst decomposition in homogeneous Pd systems is a study by Kappe and co-workers (Scheme 2) [34]. Pd black formation was observed in the development of a protocol for a Pd-catalyzed oxidative cleavage of olefins to their corresponding aldehydes and ketones. The system was studied in a relatively simple and cost-effective perfluoroalkoxy (PFA) coil. Improved yields could be obtained by using pure O_2_ instead of air. Poly(ethylene glycol)-400 (PEG-400) was utilized as a co-solvent in an attempt to stabilize the Pd catalyst under process intensified conditions. PEG has received significant attention as an inexpensive, non-volatile, and an environmentally benign solvent. Visual inspection and inductively coupled plasma mass spectrometry (ICPMS) analysis demonstrated that virtually no Pd black formation occurred when PEG-400 was used as co-solvent. Catalyst loading was lowered successfully to 0.1 mol % without compromising product yield. A variety of alkenes were converted in moderate to good yields using the flow protocol.

**Scheme 3:**
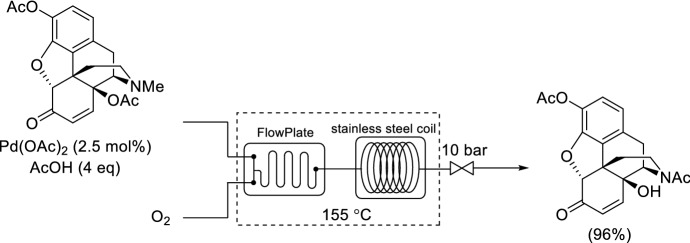
Continuous flow oxidative *N*-demethylation of 14-hydroxymorphinone 3,14-diacetate

The *N*-methyl group is contained in naturally occurring alkaloids (e.g., morphine, codeine, thebaine or oripavine) and its removal is needed to gain access to potent *N*-alklyated opioid receptor antagonists. In particular, the Pd-catalyzed aerobic *N*-demethylation of 14-hydroxymorphinone 3,14-diacetate was achieved using pure O_2_ in a 100 mL stainless steel flow reactor on a 1 kg scale (Scheme 3) [35]. Prior to scale-up, micro-calorimeter (μRC) experiments and differential scanning calorimetry (DSC) had demonstrated that a safe operation can be ensured. The reaction could be successfully scaled-up by selecting a proper structure geometry for the gas–liquid mixing function [36]. The combination of a FlowPlate A6 and a coiled tube provided good mixing of the gas and liquid phases and sufficient residence time, respectively, for almost quantitative conversion. Subsequent hydrogenation and hydrolysis in flow resulted in noroxymorphone—a precursor to naloxone used to block the effects of opioids in the case of overdose.

**Scheme 4:**
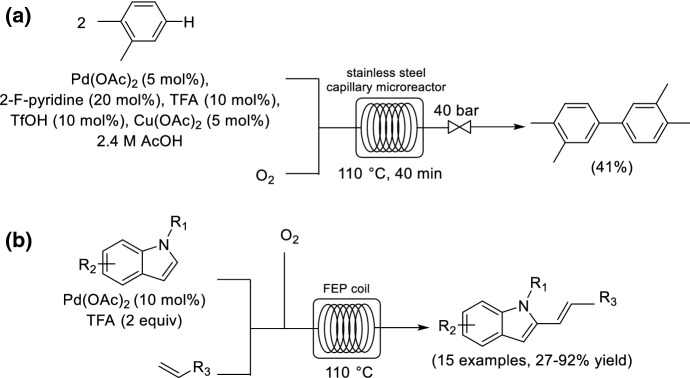
Continuous flow synthesis for **a** cross-dehydrogenative coupling of the unactivated arene *o*-xylene to 3,4,3′,4′-tetramethyl-biphenyl and **b** cross-dehydrogenative Heck reaction of indoles and alkenes

The American Chemical Society (ACS) Green Chemistry Institute (GCI) Pharmaceutical Roundtable identified the direct activation of an aryl hydrogen (C–H activation), the conversion of Ar–H into Ar–Ar, as one of the top aspirational reaction classes [37]. One of the benefits of developing selective C–H activation procedures is that it avoids the preparation of aryl halides. An interesting example that highlights the use of process intensified conditions under continuous flow for C–H activation is the aerobic cross-dehydrogenative homocoupling of the unactivated arene *o*-xylene to 3,4,3′,4′-tetramethyl-biphenyl [38]. The product is important since it is used as a precursor for metal organic frameworks (MOFs). Stahl and co-workers reported a Pd-catalyzed batch approach using 1 bar O_2_ under unoptimized batch conditions to give the product in a very low yield (7%) after 17 h reaction time [39]. Noël and co-workers successfully developed conditions that were amenable to flow processing. The reaction time could be reduced to 40 min by operating at 100 °C and 40 bar within a stainless steel capillary microreactor to afford the product in 41% yield (Scheme 4a), albeit with higher catalyst and additive loadings.

The same group also reported the connection of two different C–H bonds via a cross-dehydrogenative Heck reaction of indoles and alkenes to prepare vinylindoles [40]. A small library of 3-vinylindole derivatives was prepared in residence times between 10 and 20 min under continuous flow conditions (Scheme 4b).

**Scheme 5:**
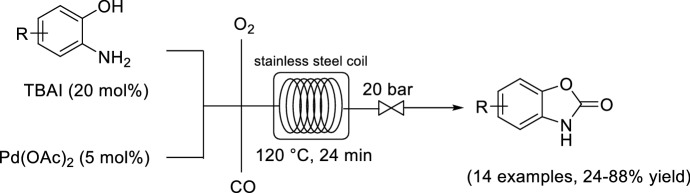
Continuous flow oxidative carbonylation

A benefit of continuous flow reactors is the ability to precisely control the gas stoichiometric ratio when using multiple gas feeds [41]. Kappe and co-workers reported the development of a Pd-catalyzed oxidative carbonylation for the formation of carbonylated heterocycles by using CO and O_2_ (Scheme 5) [42]. However, the composition of CO in O_2_ between 15.5 and 93.9 vol% is within the explosive regime. Typically, batch reactions are operated outside of this regime to ensure safety. The flow experiments demonstrated that the stoichiometric ratio of CO to O_2_ had a critical influence on the yield. A high concentration of CO is important for the carbonylation; however, too much CO was determined to cause faster deactivation of the Pd(II) catalyst by reduction to Pd(0). O_2_ is also critical to the reaction because it maintains a high level of iodine, which is critical for the reoxidation of Pd(0) to Pd(II), although too much O_2_ can oxidize the substrate. The optimal CO to O_2_ ratio was identified as 1:1, which is within the explosive regime but the characteristics of the flow set-up enabled operation within this regime, which would otherwise be inaccessible under batch conditions.

### Cu-Catalyzed Reactions

**Scheme 6:**
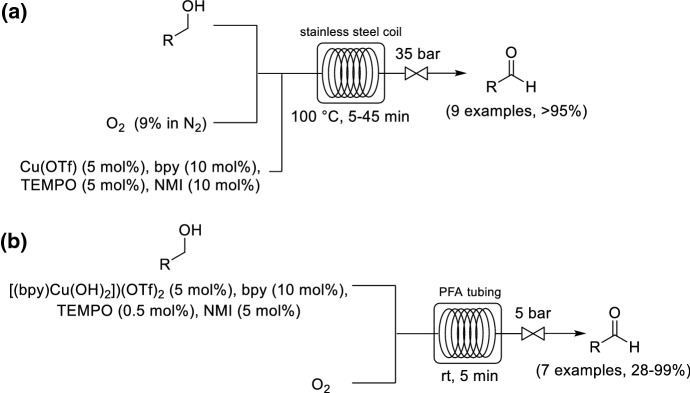
Continuous flow Cu-catalyzed aerobic oxidation of alcohols to aldehydes by using **a** diluted O_2_ and **b** pure O_2_

Copper is a non-noble and inexpensive abundant metal, thus its use as a catalyst for aerobic oxidations is desirable [43]. Stahl and co-workers developed a continuous flow process for the aerobic oxidation of alcohols using a Cu(I)/TEMPO catalyst system and 9% O_2_ in N_2_ (Scheme 6a) [44]. The reaction rate in this system is usually limited by the aerobic oxidation of Cu(I) to Cu(II). One strategy to increase the O_2_ concentration in the liquid phase to accelerate the oxidation of Cu(I) is through increasing the system pressure. Thus, relatively short residence times could be achieved by operating at 35 bar pressure and 100 °C to oxidize a variety of alcohols to their corresponding aldehydes. Longer residence times were used for less reactive alcohols. The flow protocol was applied to the oxidation of benzyl alcohol to benzaldehyde, with 100 g of product synthesized over a 24 h operation time.

As stated above in the section Using Diluted O_2_, a limitation of using O_2_ diluted with N_2_ is that the O_2_ is competing with N_2_ for dissolution in the liquid phase. Favre-Réguillon and co-workers studied the same Cu(I)/TEMPO alcohol oxidation but used pure O_2_ as the oxygen source (Scheme 6b) [45]. They argued that by utilizing pure O_2_ it would be possible to operate the system at a lower pressure and decrease the reaction temperature, because the reaction would be less likely to be mass transfer limited. A substantially enhanced reaction rate and similar yields were achieved by using higher concentrations of O_2_. The same residence time could be used to obtain similar yields at 5 bar pressure and room temperature. Superior space time yields (i.e., the product yield per unit of time and per reactor volume) were achievable by using pure O_2_ when compared to using diluted O_2_.

### Miscellaneous

**Scheme 7a,b:**
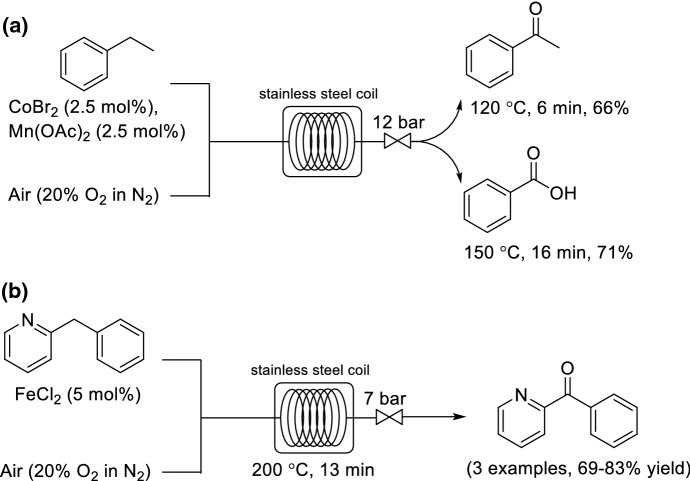
Continuous flow synthesis. **a** Oxidation of ethylbenzene to acetophenone/benzoic acid. **b** Fe-catalyzed aerobic oxidation of 2-benzylpyridines

One of the most active liquid phase oxidation systems is cobalt, manganese and bromide salts in acetic acid as solvent (MC-system). Kappe and co-workers investigated the oxidation of ethylbenzene to acetophenone by using either hydrogen peroxide (H_2_O_2_) or air (Scheme 7a) [46]. In contrast to when using H_2_O_2_ as oxidant, no catalyst deactivation was observed for oxidations using O_2_. The selectivity of reaction for either acetophenone or benzoic acid could be controlled through careful manipulation of the residence time and reaction temperature, thus demonstrating the benefit of having precise control over the reaction parameters within a flow environment. A short residence time (6 min) and low reaction temperature (120 °C) resulted in acetophenone as the main product, whereas a long residence time (16 min) and high reaction temperature (150 °C) resulted in the formation of benzoic acid as the main product. The reaction times were significantly shorter than previously published examples for the aerobic oxidation of ethylbenzene (15–50 h).

By using a similar continuous flow configuration, Pieber and Kappe also developed a flow protocol for the Fe-catalyzed aerobic oxidation of 2-benzylpyrdines to their corresponding ketones (Scheme 7b) [47]. Propylene carbonate could be used as solvent instead of using environmentally less desirable DMSO. Propylene carbonate is a nontoxic and biodegradable cyclic carbonate.

**Scheme 8a,b:**
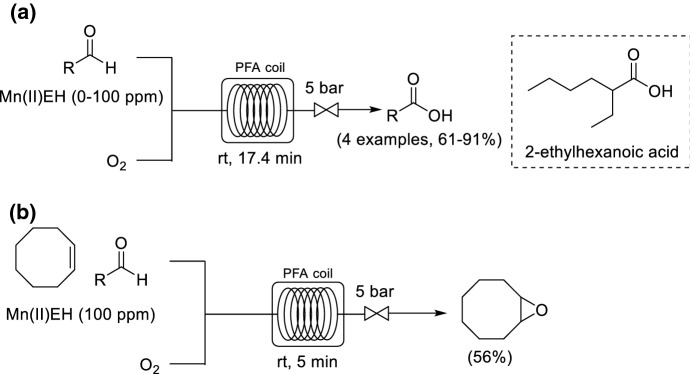
Continuous flow synthesis. **a** Uncatalyzed and catalyzed oxidation of aldehydes to carboxylic acids, **b** Mukaiyama epoxidation of *cis*-cyclooctene

Favre-Réguillon and co-workers studied the liquid phase uncatalyzed autoxidation of aldehydes to carboxylic acids within a relatively simple and cost-effective PFA coil reactor operated at room temperature and 5 bar pressure (Scheme 8a) [48]. The group successfully showed that an increase in the two-phase superficial velocity resulted in higher conversions due to the increase of the recirculation velocity within the liquid slugs. At room temperature, and in less than 2 min residence time, without using metal catalysts or radical initiators, conversions up to 50% were observed. By increasing the residence time and, in some instances, by addition of Mn(II) 2-ethylhexanoate (Mn(II)EH) as a catalyst at 100 ppm, conversions could be improved to > 95%. In a later study, the same group conducted a high-throughput optimization for the oxidation of 2-ethylhexanal to 2-ethylhexanoic acid, one of the acids with the highest production capacity worldwide, using a sequential pulse experimentation approach to minimize material consumption [49]. Through this study, the group demonstrated the synergistic use of a large range of salts and Mn(II) catalyst resulted in highly selective aldehyde oxidation. The optimization provided conditions that afforded ethylhexanoic acid in 98% yield within 6 min residence time. In a subsequent final optimization and scale-up study, the oxidation of 2-ethylhexanal in flow was achieved successfully at a throughput of 130 g/h [50]. The same group investigated a Mukaiyama epoxidation of *cis*-cyclooctene by using a similar flow configuration (Scheme 8b) [51].

**Scheme 9:**
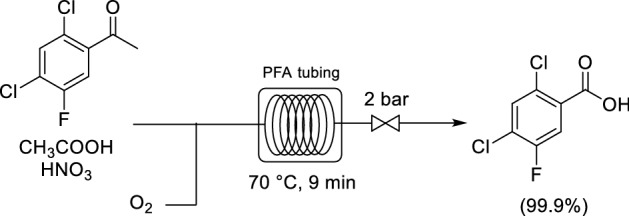
Continuous flow oxidation of 2,4-dichloro-5-fluoroacetophenone

Yu and co-workers developed a continuous flow synthesis for the oxidation of 2,4-dichloro-5-fluoroacetophenone to 2,4-dichloro-5-fluorobenzoic acid with air and pure O_2_ [52]. 2,4-Dichloro-5-fluorobenzoic acid is a very important API intermediate. Nitric acid functioned as a catalyst and co-solvent (Scheme 9). The reaction proceeded under 2 bar pressure and at 70 °C; thus, only standard PFA tubing was required. The reaction proceeded to give a quantitative yield and the system was stable for 1 kg product to be manufactured. Air could also be utilized as the oxygen source, with only a small drop in yield observed.

## Heterogeneous Catalysis

A limitation of homogeneous catalysis is that the product needs to be separated from the catalyst after the reaction. The use of a heterogeneous catalyst is one method to prevent the product from becoming contaminated by the catalyst because it is in a different phase, provided that leaching into the liquid phase does not occur. The active metal is dispersed on a support, such as carbon, metal oxide or other inorganic material as a packed bed within a flow system [53]. A number of techniques are used for the preparation of catalysts, including impregnation, adsorption, precipitation or ion exchange [54]. The stabilization of a catalyst on an inert solid support can also improve the thermal stability of catalysts. The improved thermal stability is particularly beneficial given the high temperatures often employed within continuous flow reactors. The incorporation of one or more promoters, derived from the early transition metals, lanthanides and/or main group elements, can further modulate activity and selectivity. However, additional challenges exist for heterogeneous catalyst systems compared to their homogeneous counterparts. Isothermal temperature control can be difficult to obtain and the efficient mixing between the gas, liquid and solid phases can be difficult to achieve [28]. A high steady-state conversion is sometimes not possible to achieve due to catalyst deactivation and/or leaching [55]. An additional difficulty regarding their widespread uptake is that the preparation of heterogeneous catalysts is often outside the skill set of a standard organic chemist.

**Scheme 10a–c:**
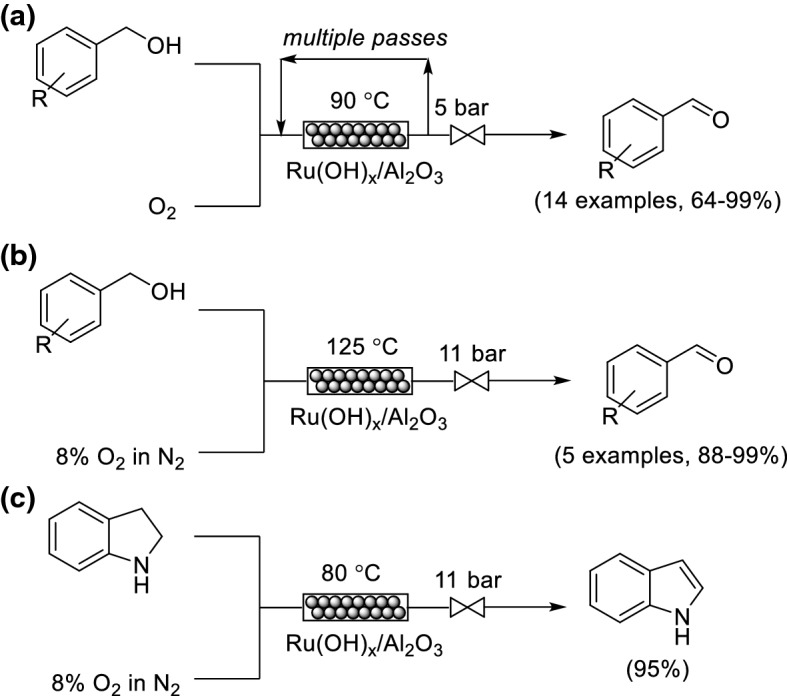
Flow oxidations using Ru(OH)_*x*_/Al_2_O_3_ as a packed bed. **a** Alcohol oxidation using a recirculating strategy. **b** Alcohol oxidation from a single pass. **c** Dehydrogenation of indoline to indole

The aerobic oxidation of alcohols using transition metal catalysts on solid supports has received significant attention [56]. Hii and co-workers incorporated a heterogeneous Ru(OH)_*x*_/Al_2_O_3_ catalyst within an adapted version of the X-Cube flow reactor for the oxidation of benzylic and allylic alcohols using pure O_2_ (Scheme 10a) [57]. The system could be considered inherently safe under the conditions used, because, even under the maximum pressure of 25 bar, only −97.3 J heat can be generated from the process based on the amount of O_2_ present. This amount of heat corresponds to an adiabatic temperature rise, Δ*T*_ad_ = 77 °C, and pressure rise, Δ*P* = 7.1 bar, from the liberation of CO_2_, a temperature and pressure rise that the reactor can safely withstand. High yields were obtained; however, the flow system was essentially operated in a semi-batch manner, because the reacting mixture was recirculated continuously through the packed bed reactor to achieve high conversions. The resulting reaction times were between 0.75 h to 7 h for the different substrates. Stahl and co-workers also reported using a heterogeneous Ru(OH)_*x*_/Al_2_O_3_ catalyst for the aerobic oxidation of alcohols (Scheme 10b) [58]. However, in this case, a diluted oxygen source (8% O_2_ in N_2_) was used. The catalyst deactivation kinetics were characterized to provide a basis for identification of process conditions that enabled high single-pass yields for a number of aldehydes. In particular, the oxidation of 2-thiophenemethanol was achieved in > 99% yield, which was successfully maintained over a 72 h operation time. The same flow system was also applied to the dehydrogenation of indoline to indole (Scheme 10c).

**Scheme 11:**
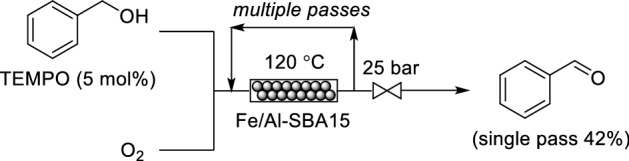
Continuous flow oxidation of benzyl alcohol using Fe/Al-SBA15 as a packed bed

Kappe and co-workers reported the selective oxidation of benzyl alcohol to benzaldehyde by using an iron oxide nanoparticle catalyst stabilized in a mesoporous aluminosilicate support (Fe/Al-SBA15) within a continuous flow reactor, which the authors term as “flow-nanocatalysis” (Scheme 11) [59]. A 42% fraction of benzyl alcohol could be oxidized within a single pass but recirculation was necessary to achieve full conversion. ICPMS analysis demonstrated that the catalyst does not leach from the reactor, thus indicating the heterogeneity of the reaction mechanism.

**Scheme 12:**
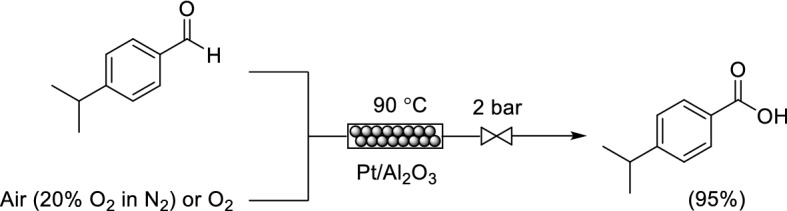
Continuous flow oxidation of 4-isopropylbenzaldehyde to cumic acid using Pt/Al_2_O_3_ as a packed bed

Jensen and co-workers studied the oxidation of 4-isopropylbenzaldehyde to cumic acid, an important API intermediate, using a Pt/Al_2_O_3_ packed bed within a silicon–Pyrex microreactor (Scheme 12) [60]. An aqueous slurry of the catalytic material was loaded onto glass beads to prepare Pt/Al_2_O_3_ as a tightly packed catalyst bed. Conditions that enabled air to be used instead of oxygen without compromising yield and selectivity were identified successfully. The transformation was estimated to take only a few seconds under continuous flow conditions, compared to several hours under semi-batch conditions. Very recently, Lee and co-workers reported the oxidation of cinnamaldehyde to cinnamic acid using silica supported Pt nanoparticles under base-free continuous flow conditions [61].

**Scheme 13:**
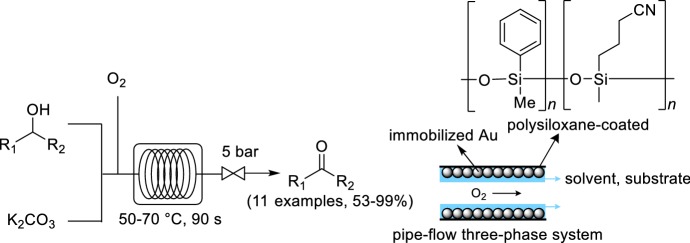
Gold-coated microchannels for the oxidation of alcohols

A gold catalyst immobilized within a microreactor was used for the oxidation of alcohols with O_2_ by Kobayashi and co-workers (Scheme 13) [62]. A polysiloxane-coated capillary column was used. The cyanopropyl groups of the polysiloxane were reduced to primary amines, which were cross-linked to the gold catalyst by passing through a colloidal solution of microencapsulated gold at 170 °C for 5 h. The optimal conditions for the oxidation of alcohols were identified as ~ 60 °C, 5 bar pressure and 90 s residence time. A pipe-flow three-phase system was obtained under these conditions, which provided very good mixing between the gas, liquid and solid phases. Benzylic, aliphatic and allylic alcohols were all converted to their corresponding aldehyde or ketone in good to excellent yields. The system was operated successfully using 1-phenylethanol as substrate for 4 days without loss in activity, and no leaching of gold was observed.

**Scheme 14:**
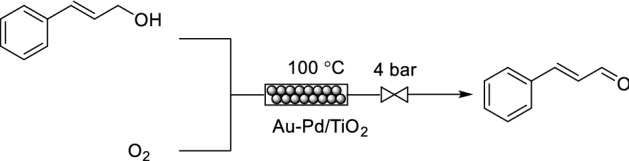
Flow oxidation of cinnamyl alcohol to cinnamaldehyde using Au–Pd/TiO_2_ as a bimetallic packed bed catalyst

The activity, selectivity and stability of a catalyst can be improved through the development of bimetallic catalysts due to the synergistic effects between the two metals when compared to their monometallic counterparts [63]. Gavriilidis and co-workers reported the oxidation of cinnamyl alcohol to cinnamaldehyde by using a bimetallic catalyst system, Au–Pd/TiO_2_, as a packed bed within a capillary microreactor system (Scheme 14) [64]. The catalyst was prepared by co-impregnation, perhaps the simplest approach for catalyst bimetallic catalyst preparation, with an Au–Pd weight ratio of 1:19. Initially, the researchers demonstrated that the catalyst was stable over a 30 h time period for the oxidation of benzyl alcohol to benzaldeyde. For the cinnamyl alcohol oxidation, in addition to cinnamaldehyde formation, 3-phenyl-1-propanol and trans-*β*-methylstyrene were also observed as side products. An improved selectivity for cinnamaldehyde was observed at higher oxygen equivalents, albeit with elevated catalyst decomposition. Elevated reaction temperatures were also responsible for catalyst deactivation. A partial recovery in catalyst activity could be achieved by treatment with hydrogen.

**Scheme 15a,b:**
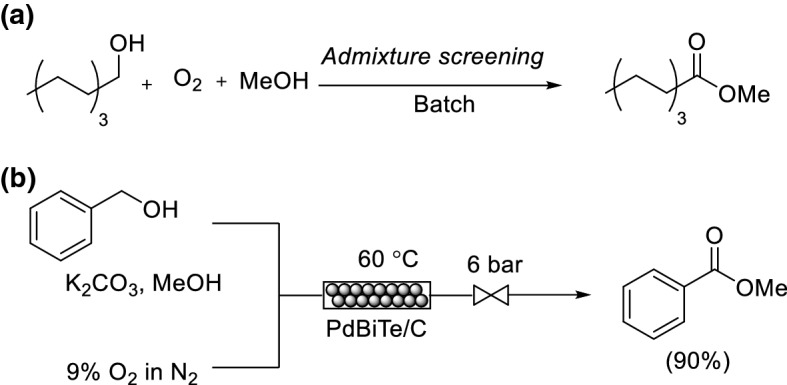
Multicomponent catalysts for aerobic oxidation. **a** Admixture screening for oxidation of 1-octanol. **b** Continuous flow oxidation of benzyl alcohol using a multicomponent catalyst

A key challenge to designing sustainable flow processes is the discovery of robust multicomponent catalysts which display high catalyst turnovers. The inclusion of promoters has been associated with mediating the adsorption and dissociation of O_2_, thus preventing over oxidation of the metal surface. Stahl and co-workers conducted an admixture screening in batch for the discovery of new heterogeneous Pd catalyst and promoter compositions [65]. Over 4000 catalyst compositions were explored for the oxidative methyl esterification of 1-octanol to methyl octanoate (Scheme 15a). The screening of simple binary and ternary admixtures of Pd/charcoal in combination with one or two metal and/or metalloid components was conducted. The optimal results were observed with Bi-, Te- and Pb-based additives. PdBi_0.35_Te_0.23_/C as catalyst was utilized within a flow system for the oxidative methyl esterification of benzyl alcohol (Scheme 15b). There was no drop in catalytic activity over 120 h after nearly 60,000 catalytic turnovers. ICP-AES (inductively coupled plasma atomic emission spectroscopy) analysis to determine metal content showed that less than 1 ppm (part-per-million) of the three elements leached from the packed bed, corresponding to a stoichiometry change of PdBi_0.35_Te_0.21_ to PdBi_0.21_Te_0.12_.

## Uncatalyzed Reactions

**Scheme 16a–c:**
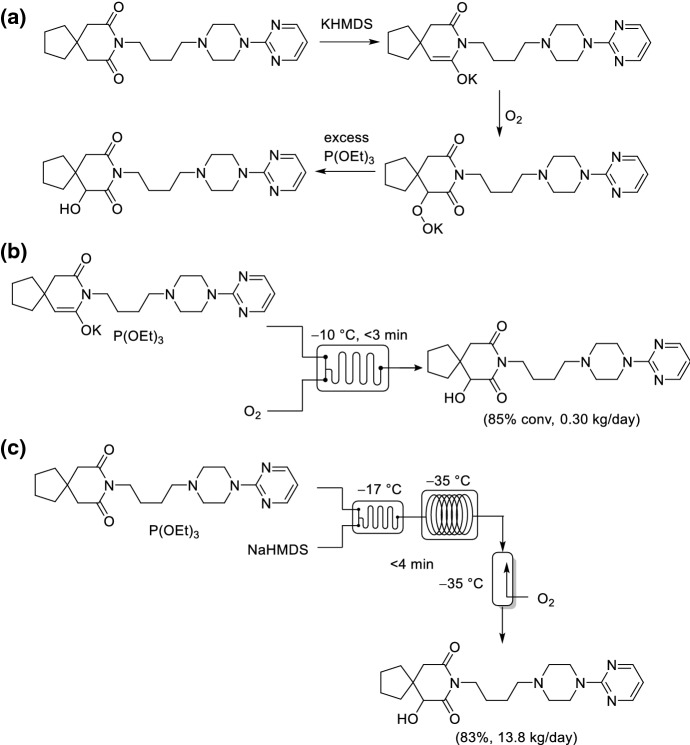
Aerobic oxidation of buspirone to form 6-hydroxybuspirone. **a** Reaction scheme. **b** Stacked-microreactor configuration. **c** Trickle bed reactor setup

The earliest example of a translation of a batch manufacturing process to flow for an aerobic oxidation within the pharmaceutical sector was described by Bristol–Myers Squibb (BMS). BMS researchers were interested in the oxidation of the imide within buspirone to produce 6-hydroxybuspirone (Scheme 16a). Initially, they reported the aerobic oxidation in batch to produce 6-hydroxybuspirone at a 10 kg scale [66]. The reaction was achieved in ~ 71% yield with a throughput of 7.53 kg day^−1^. However, the conditions used were sub-optimal, the reaction was conducted using synthetic air (6% O_2_ in N_2_) to ensure the headspace was kept below the LOC, and cryogenic conditions (− 78 °C) were used to minimize mass transfer effects and to control the reaction exotherm (Δ*H* = 685 kJ mol^−1^, Δ*T*_adiabatic_ = 68 °C). The poorer mass transfer observed at larger scales caused the reaction time to increase from 8 h within a laboratory setting to 16–24 h at the pilot plant scale. Pure O_2_ had been demonstrated to significantly increase the reaction rate. Thus, BMS sought to identify conditions under continuous flow that could be used to prepare large quantities [67]. Initial studies were conducted using pure O_2_ at an elevated temperature of −10 °C within a CPC CYTOS stacked-plate microreactor (Scheme 16b). A higher cooling efficiency was possible from the high reactor surface-to-volume ratio within the microreactor. A > 85% conversion could be achieved in less than 3 min residence time to achieve a throughput of 300 g day^−1^, which, unfortunately, was insufficient to reach manufacturing demands and was much lower than the batch manufacturing protocol. Subsequently, a trickle bed reactor system was developed, which was operated at −38 °C and < 4 min residence time with a counter current flow of O_2_ (Scheme 16c) [67]. Four reactors in parallel through a numbering up strategy enabled the production of 15.8 kg day^−1^, which was sufficient throughput for late stage clinical trials. The improvement in yield and throughput from the batch process was attributed to the higher O_2_ mass transfer rate and the utilization of pure O_2_ instead of a diluted O_2_ blend.

**Scheme 17:**
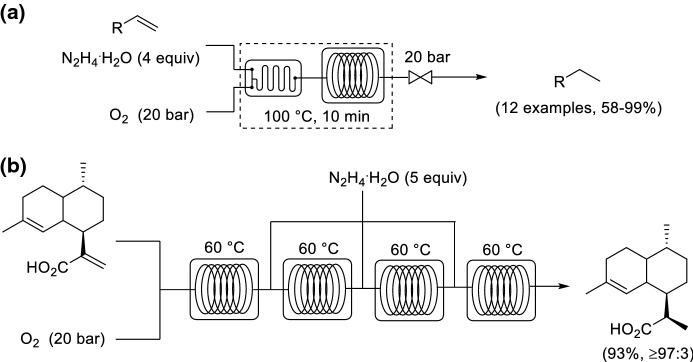
Continuous flow in situ generation of diimide (HN=NH) by O_2_ for the reduction of alkenes applied to **a** highly reactive alkenes and **b** a multi-injection strategy for the reduction of artemisinic acid

The catalyst-free in situ generation of diimide (HN=NH) as a hydrogenation transfer agent for the reduction of alkenes was achieved through the oxidation of hydrazine monohydrate by using O_2_ as oxidant. Kappe and co-workers reported a continuous flow protocol (Scheme 17a) [68]. However, it was difficult to drive the reaction to full conversion in the case of poorly reactive alkenes. An important step in the production of artemisinin is the diastereoselective reduction of artemisinic acid to dihydroartemisinic acid. A commercial scale synthesis in batch using synthetic air (5% O_2_ in N_2_) under atmospheric pressure was reported on a 1 kg scale by Sanofi-Aventis. The reaction proceeded in 11 h using 3 equivalents of hydrazine monohydrate to afford more than 90% yield and 97% diastereoselectivity, corresponding to a space time yield (STY) of 0.023 mmol L^−1^ h^−1^ [69]. Kappe and co-workers devised a multi-injection strategy for the introduction of fresh hydrazine hydrate for the reduction of poorly reactive alkenes. (Scheme 17b) [70]. The synthesis of dihydroartemisinic acid could be achieved in 93% yield, corresponding to a STY of 0.56 mmol L^−1 ^h^−1^. The flow procedure provided a significant improvement in space–time yield in comparison to the batch synthesis. The reduction of thebaine was also achieved using a multi-injection approach but by using four inlet feeds for hydrazine monohydrate [71].

## Organomagnesium and Organolithium Reagents

**Scheme 18:**
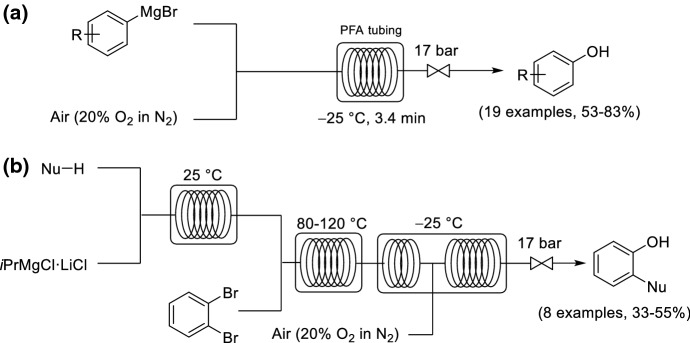
Continuous flow protocols for **a** direct oxidation of aryl Grignards to form substituted phenols; **b** in situ formation of aryl Grignards and subsequent oxidation

He and Jamison investigated the direct oxidation of aryl Grignard reagents to form substituted phenols by using compressed air within a segmented flow reactor (Scheme 18a) [72]. Typically, under batch conditions, the aerobic oxidation of aryl Grignard reagents is poor yielding (10–20%) and a mixture of by-products are formed. The flow protocol was successful for the formation of a range of phenols, with either electron-withdrawing or -donating groups, and tolerant for a range of oxidation sensitive functional groups, including alkenes, amines, and thioethers. The protocol was then extended to a three-step continuous flow process by an upstream in situ generation of the aryl magnesium species starting from 1,2-dibromobenzene, *i*PrMgCl·LiCl and a nucleophile (Scheme 18b). Benzynes were formed as intermediates from the dibromobenzene and the isopropylmagnesium chloride, and subsequent addition of the nucleophile gave the arylmagnesium species. Aerobic monooxygenation finally afforded the phenols in moderate yields after a combined residence time of 14 min for all the steps.

**Scheme 19:**
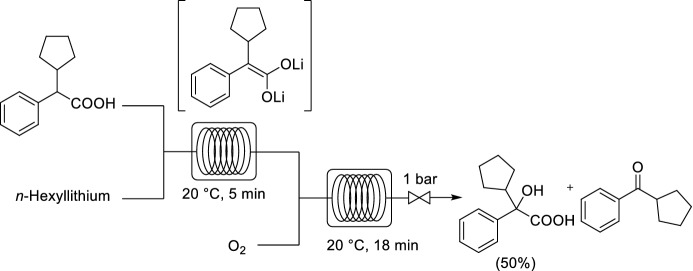
Continuous flow *α*-lithiation and aerobic oxidation to synthesize cyclopentyl mandelic acid

Cyclopentyl mandelic acid (CMPA), a key intermediate in the synthesis of glycopyrronium bromide (glycopyrrolate), is a synthetically challenging intermediate to prepare. The existing manufacturing routes utilize Grignard reagents to afford CPMA in 28–56% yield. Kappe, Luisi and co-workers devised a flow protocol for the sequential *α*-lithiation and subsequent hydroxylation of *α*-phenylcyclopentylacetic acid by aerobic oxidation (Scheme 19) [73]. Hexyllithium was utilized as a cost-effective and industrially safe base. A multistep continuous flow protocol was developed that afforded CPMA in 50% isolated yield under homogeneous and mild conditions (atmospheric pressure and room temperature). However, it was difficult to further improve this process and very challenging to prevent decomposition of the desired product, with excess O_2_ resulting in the formation of the undesired ketone product. The reaction was very sensitive to changes in the input parameters, including temperature, pressure and residence time, thus demonstrating the importance of having precise control over the reaction parameters.

## Membrane Technologies

A membrane acts as a gas permeable contact interface between the liquid phase and gas phase to enable the liquid phase to be saturated with dissolved gas [74]. Membranes have been used to pre-load the liquid phase with O_2_ before a reaction or to continuously introduce O_2_ to the liquid phase throughout the duration of a reaction. The nature of the contacting method ensures the process is inherently safe because the liquid phase and gaseous O_2_ are in different channels, thus flammable organic solvents are never in the presence of gaseous O_2_. In addition, this method also enables better control over residence time at different gas flow rates compared to flow regimes containing undissolved O_2_, such as a segmented gas–liquid flow regime, within a single microchannel. Membranes are designed to have a large interfacial area to minimize mass transfer effects.

### Tube-in-Tube Reactor

**Scheme 20:**
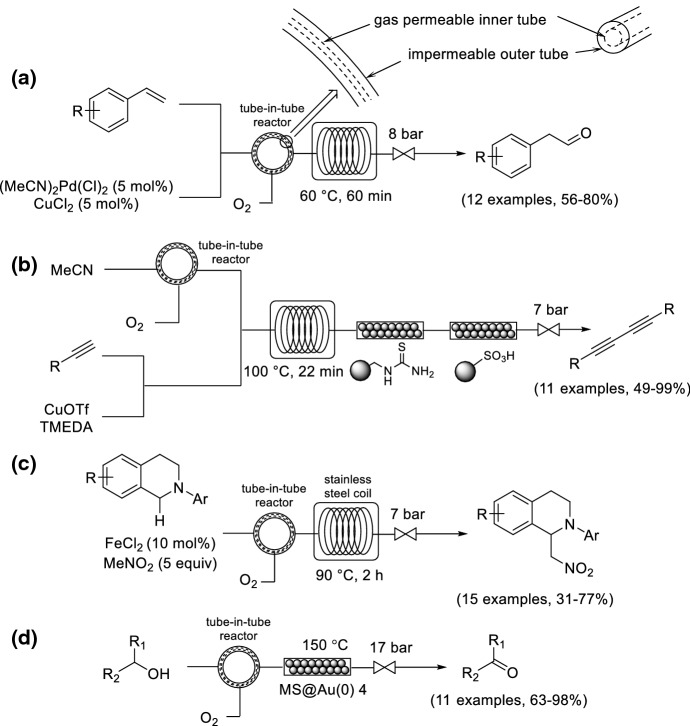
Continuous flow oxidation using a tube-in-tube reactor as the gas addition module

Ley and co-workers pioneered the tube-in-tube reactor gas-loading concept to enable the safer introduction of gases into the liquid-phase [75]. A semipermeable polymeric membrane is permeable to gases but impermeable to liquids. The nature of the contacting method ensures that the use of pure O_2_ is inherently safe, because it avoids the direct mixing of gaseous O_2_ with the liquid phase. Teflon AF-2400 (a fluoropolymer) was selected as an appropriate membrane material due to its high permeability to a wide range of gases while remaining impermeable to (nonfluorinated) liquids, its thermal stability and for its resistance to corrosion. The tube-in-tube reactor was successfully demonstrated by Ley and co-workers on the synthesis of phenylacetaldehydes from functionalized styrenes using an aerobic anti-Markovnikov Wacker oxidation involving pure O_2_ (Scheme 20a) [76]. The O_2_ was loaded into the reaction mixture using the tube-in-tube gas-loading tool, and the gas-saturated solution then passed through a tubular reactor at 60 °C for 60 min. A copper-catalyzed Glaser–Hay acetylene homocoupling reaction was also demonstrated by the same group (Scheme 20b) [77]. The reaction mixture was loaded with the tube-in-tube gas-loading tool, and the gas-saturated solution then passed through a tubular reactor at 100 °C. The copper and the amine base were then removed from the flow stream by passing it through a cartridge of polymer-supported thiourea and polymer-supported sulfonic acid. The 1,3-butadiynes were isolated in up to quantitative yields, generally without the need for chromatography. Polyzos and co-workers translated the nitro-Mannich *α*-C(sp^3^)–H functionalization of *N*-aryl tetrahydroisoquinolines mediated by iron salts to flow by using the tube-in-tube reactor for the introduction of O_2_ (Scheme 20c) [78]. Kirschning and co-workers used a Teflon AF-2400 tube-in-tube reactor to pre-saturate the liquid feed with O_2_ before the feed entered a packed-bed reactor containing immobilized gold-doped nanoparticles (Scheme 20d) [79]. Au-NPs were immobilized on a nanostructured Fe_3_O_4_-containing core and a silica shell that was heated in an external oscillating electromagnetic field to catalyze benzylic and allylic alcohol oxidation in the presence of pure O_2_ or air.

**Scheme 21a,b:**
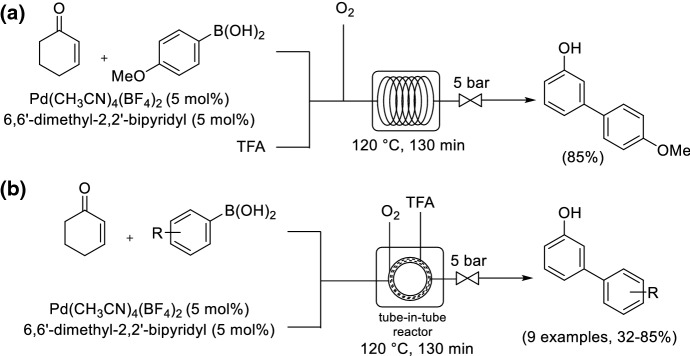
Complementary microreactors for a sequential Pd-catalyzed oxidative Heck/dehydrogenation. **a** Segmented capillary flow system. **b** Tube-in-tube reactor

Park and co-workers reported the study of the synthesis of meta-substituted phenols via an oxidative Heck/dehydrogenation sequence within two complementary microreactors, a segmented flow capillary system and a tube-in-tube microreactor [80]. The capillary segmented flow reactor had an internal volume of 0.098 mL, which enabled a microgram optimization study without wasteful reagent consumption (Scheme 21a). The reaction took 130 min within a segmented flow regime compared to 36 h in a traditional batch system. The conditions were then successfully translated to gram scale by using a larger volume tube-in-tube microreactor (7.72 mL) (Scheme 21b). The yields for the reaction of 4-methoxyphenylboronic acid and cyclohex-2-enone conducted within a segmented flow regime and tube-in-tube reactor were comparable, 85% and 84%, respectively.

**Scheme 22:**
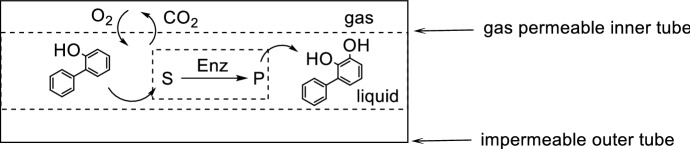
Tube-in-tube reactor for a biocatalytic hydroxylation

The performance of reactions can be improved by the continuous supply of O_2_ through the membrane during the reaction, thus replenishing O_2_ as it is consumed, rather than pre-saturating the liquid feed prior to the reaction. Continuous penentration of gas ensures that the liquid phase is saturated with O_2_ throughout the reaction. Enzyme-mediated oxidation chemistry is an emerging topic in organic synthesis [81]. Woodley and co-workers utilized a tube-in-tube flow reactor for the collection of time-series experimental data for the biocatalytic oxidation of glucose to glucose-δ-lactone by glucose oxidase (Scheme 22) [82]. The system displayed a low-dispersed flow regime at the scale investigated resulting in a high degree of accuracy for the kinetic data with minimal material consumption. Buehler and co-workers investigated the hydroxylation of 3-phenylcatechol catalyzed by 2-hydroxybiphenyl 3-monooxygenase (Scheme 22) [83]. The tube-in-tube reactor performs well for research scale experimentation; however, the technology can suffer from limited scalability in terms of performance at scale-up due to mass transfer limitations [84].

**Scheme 23:**
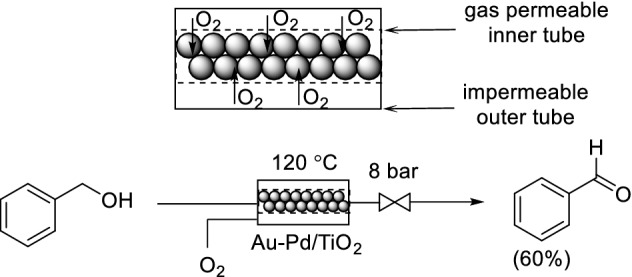
Catalyst immobilization within a tube-in-tube reactor for benzyl alcohol oxidation

A further benefit of the tube-in-tube approach is that catalyst immobilization is possible within the tube containing the liquid phase. Gavriilidis and co-workers packed a porous inner tube of a Teflon-2400 tube-in-tube reactor directly with the bimetallic catalyst Au–Pd/TiO_2_ (discussed earlier). The flow system was applied to the oxidation of benzyl alcohol to benzaldehyde (Scheme 23) [85]. Again, the benefit here is that the O_2_ is introduced along the whole of the packed bed, thus preventing the system from becoming starved of oxygen towards the end of the reactor. Subsequently, the same group developed a packed-bed porous ceramic membrane reactor [86]. However, results indicated that the rate of O_2_ supply may not be sufficient for fast reactions not to be mass transfer limited [87].

### Tube-in-Shell Reactor

**Scheme 24:**
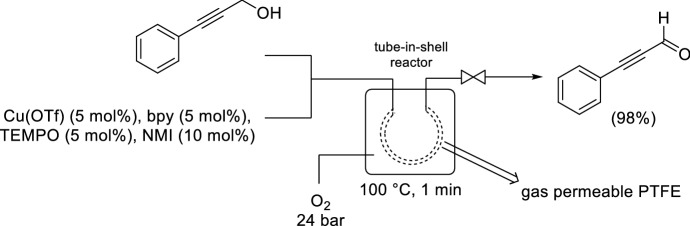
Tube-in-shell reactor for aerobic oxidations

Stahl and co-workers reported a tube-in-shell reactor (also referred to as tube-in-flask reactor) as an alternative membrane strategy for the safe introduction of O_2_ into the liquid phase (Scheme 24) [88]. Gaseous O_2_ is introduced from a cylinder into a stainless steel shell and the O_2_ permeable tubing carrying the liquid phase is coiled within the shell. A limitation of the Teflon AF-2400 membrane used in the tube-in-tube applications is its relatively high cost (US $25,000/kg). The group identified a polytetrafluoroethylene (PTFE) membrane as an inexpensive alternative (US $2–10/kg) to Teflon AF-2400, which was also compatible with elevated temperatures and pressures. The system was initially demonstrated using the homogeneous Cu(I)/TEMPO catalyst system for alcohol oxidation, with good yields achieved within 1 min residence time. In this case, however, pure O_2_ was utilized due to the inherent safety associated with the introduction of O_2_ by using a membrane system. Scale-up was proposed by numbering-up the membrane reactor by assembling a configuration with multiple tubes operating in parallel. The permeable tubing was also packed with Ru(OH)_*x*_/Al_2_O_3_ and trialled for the aerobic oxidation of benzyl alcohol to afford quantitative yield within 55 min residence time.

### Dual- and Triple- Channel Microreactor

**Scheme 25:**
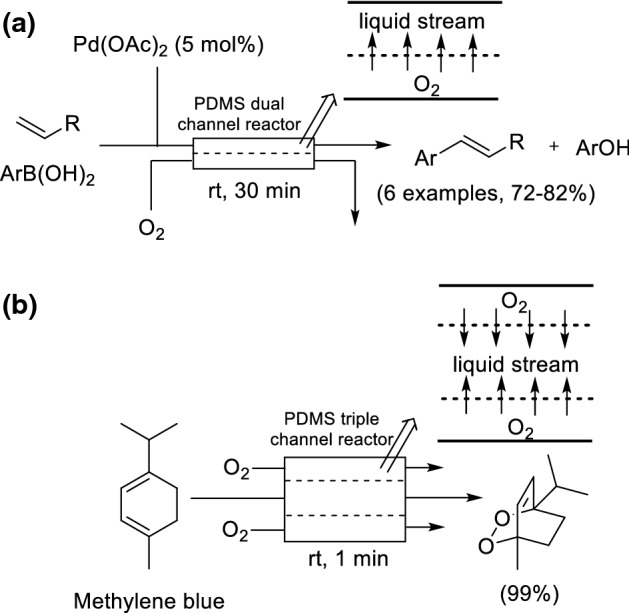
**a** Dual-channel microreactor for an oxidative Heck coupling. **b** Triple channel microreactor for photosensitized oxygenation of citronellol

Park and Kim developed a dual-channel microreactor with a thin poly(dimethylsiloxane) (PDMS) membrane sheet sandwiched between the two channels (Scheme 25a) [89]. O_2_ flowing in one channel can pass through the membrane into the second channel containing the liquid phase. The reactor system was applied to a Pd-catalyzed oxidative Heck reaction. The system was shown to give significant improvements over a batch reactor and a segmented microreactor; in particular, less Pd black formation was observed thus demonstrating improved phase contact. The cross coupling products were synthesized in good yields (72–82%) in a short 30 min residence time, compared to 12 h in batch. The dual channel microreactor was also modified and applied to a photosensitized oxygenation of (−)-citronellol [90]. The limitation of the dual-channel microreactor is that only one face of the reaction channel is exposed to gas. The group subsequently developed a triple channel microreactor where the O_2_ was introduced from either side of the liquid phase to give improved performance (Scheme 25b) [91].

## Photochemistry and Singlet Oxygen

**Scheme 26a–c:**
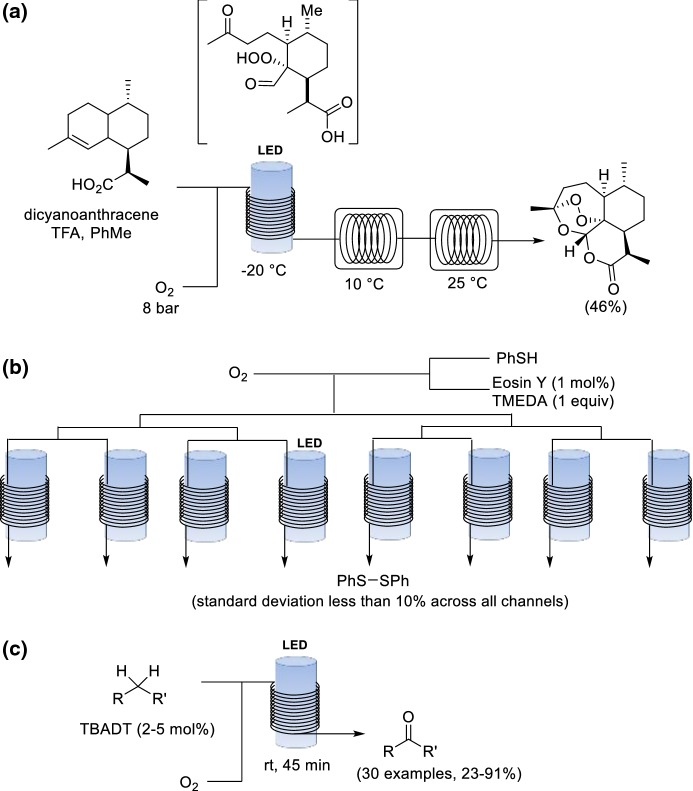
Singlet oxygen examples. **a** Multistep synthesis of artemisinin, including a photooxygenation as a key step. **b** Numbering-up of the aerobic oxidation of thiols to disulfides. **c** C(sp^3^)–H oxidation enabled by decatungstate

Singlet oxygen (^1^O_2_) is an attractive reagent due to its low cost and negligible environmental impact. The formation of ^1^O_2_ is usually achieved through the excitation of triplet oxygen (^3^O_2_) by using a photoinitator and light irradiation [92]. ^1^O_2_ is a highly reactive, unstable and explosive species, which is used as a reagent in a plethora of reactions, including heteroatom oxidations, ene reactions, and cycloaddition reactions [93]. These transformations are often thermally forbidden, but can proceed photochemically. Flow photooxygenations are treated here only briefly owing to the breadth of examples reported; comprehensive reviews can be found elsewhere [94–96]. Some examples are given in the sections below. Seeberger and co-workers developed a photochemical flow process for the preparation of ^1^O_2_ from ^3^O_2_ [97]. Subsequently, the same group applied this technology to the multistep synthesis of the anti-malarial drug artemisinin in continuous flow (Scheme 26a) [98]. The key step towards artemisinin from more readily available artemisinic acid is the formation of allylic hydroperoxide. In this step, the in situ formation of ^1^O_2_ is achieved by irradiation with blue LED light (*λ* = 420 nm), inducing an ene reaction on dihydroartemisinic acid to form a hydroperoxide as an intermediate. The accumulation of peroxide intermediates is avoided because only a small inventory of material is processed at any one time, and the intermediates are telescoped into the subsequent reaction straightaway. The addition of trifluoroacetic acid results in a Hock cleavage of the hydroperoxide. Triplet oxygen then reacts to give artemisinin. Large scale reactions utilizing ^1^O_2_ normally require non-flammable halogenated solvents to ensure safe operation, but the process could be modified in flow to use toluene owing to the low amount of oxygen present within the flow system at any one time [99].

Photochemical reactions pose an additional challenge for scale-up, because the light penetration depth has a critical influence on the performance of photooxygenations [100]. Noël and co-workers presented an interesting numbering-up approach to facilitate the scale-up of a photochemical aerobic oxidation of thiols to disulfides by placing eight capillaries in parallel that are irradiated with white LEDs (Scheme 26b) [101]. A further challenge in parallelization of gas liquid reactions is that efficient and uniform gas liquid distribution can be difficult to achieve within parallelized reactor configurations. The lack of uniform mixing in flow can cause stoichiometry imbalance and poor control of residence in the channels. In the study by Noël and co-workers, the calculated standard deviation for yield was less than 10% across the different channels.

Noël and co-workers reported a mild and selective direct oxidation of activated and unactivated C(sp^3^)–H bonds enabled by decatungstate photocatalysis (Scheme 26c) [102]. Hydrogen atom transfer (HAT) can be utilized for the production of highly reactive radical species, which can be trapped to give synthetically useful products. Decatungstate is a versatile and inexpensive HAT catalyst that readily performs hydrogen abstraction on C(sp^3^)–H upon photochemical activation. Initial optimization studies using tetrabutylammonium decatungstate (TBADT) demonstrated that full conversion could not be achieved in batch probably caused by the slow diffusion of oxygen into the liquid reaction mixture and the limited light penetration. Nonetheless, significantly improved results were observed within a continuous flow environment. In particular, the flow approach was successful for the oxidation of natural scaffolds such as (−)-ambroxide, pregnenolone acetate, (+)-sclareolide and artemisinin.

## Electrochemistry

**Scheme 27:**
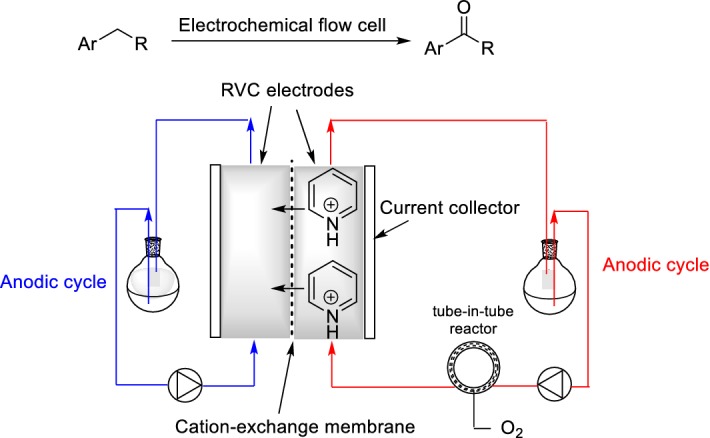
*N*-hydroxyphthalimide (NHPI)-mediated electrochemical aerobic oxidation of benzylic C–H bonds to ketones

A re-emerging area is electroorganic synthesis [103, 104]. The limitations of conventional batch electrosynthesis can be overcome by using electrochemical flow cells [105–107]. Flow electrochemical reactors can be designed to have short distances between electrodes, so no, or only low, concentrations of added supporting electrolyte are required, and a large ratio of electrode area to reactor volume exists. Mo and Jensen reported *N*-hydroxyphthalimide (NHPI)-mediated electrochemical aerobic oxidation of benzylic C–H bonds to form the corresponding ketones (Scheme 27) [108]. A tube-in-tube reactor was used for the safe introduction of O_2_ as a co-oxidant into the system. The cation-exchange membrane prevented the reductive decomposition of NHPI at the cathode, because it minimized the crossover of the NHPI anion from anolyte to catholyte. Relatively inexpensive RVC electrodes could be used instead of a platinum electrode. The system described is not inherently a continuous flow process, because the liquid feeds were recirculated to obtain high conversions to accommodate the slow reaction kinetics. In a recent perspective article, Maes and co-workers proposed that many of the challenges associated with selective aerobic oxidation on unactivated C–H bonds could be addressed effectively and safely by making use of flow electrochemistry [109].

## Green Solvents

There has been a recent drive within the pharmaceutical and fine chemicals industry towards the utilization of more environmentally benign solvents [110]. GlaxoSmithKline, among others, have published green solvent guides to support the implementation of greener solvents at the discovery and manufacturing stages in API development [111–114]. As discussed in the Introduction, the main safety challenge associated with liquid phase aerobic oxidations is the utilization of O_2_ in the presence of flammable organic solvent. A benefit of using continuous flow reactors is the ability to safely operate above the boiling point of a solvent through the pressurization of the system. This feature enables access to elevated reaction rates that would be less accessible, or even forbidden, within batch reactors. The solubility of O_2_ decreases with an increase in temperature; therefore, the ability to pressurize the system is very important. Another limitation is that the mass transfer of O_2_ from the gas to the liquid phase is often the rate-limiting step in these processes. Significant efforts have focused on identifying solvents that, under certain conditions, can dissolve all the reaction components (substrate, catalyst and O_2_) within a single phase. One such solution is to operate within the supercritical regime for a solvent to generate a single phase. CO_2_ and H_2_O are both nonflammable and have both been employed in this manner. The application of continuous flow technologies facilitates the effective and safe use of these green supercritical solvents at elevated temperatures and pressures, which would otherwise require more cumbersome, expensive and specialized batch reactors. Another way to reduce the impact of chemical manufacture is to utilize no solvent whatsoever, and to conduct reactions with neat reagents. However neat systems can add complications in terms of thermal management, impurity formation and on the physical properties of the reagents for a particular reaction. Ionic liquids and fluorous solvents are also potentially green solvents for conducting aerobic oxidations [115, 116]; however, as of yet there are no reported aerobic oxidation examples that use these solvents within continuous-flow reactors.

### Carbon Dioxide

**Scheme 28:**
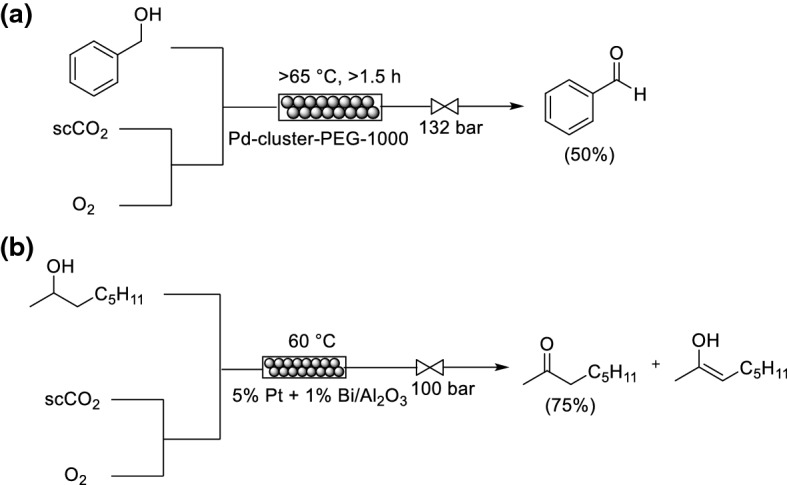
Utilization of scCO_2_ for **a** oxidation of benzyl alcohol and **b** oxidation of 2-octanol

CO_2_ has attracted significant interest as a green solvent for organic synthesis and chemical manufacture [117]. In fact, CO_2_ has been proposed as an ideal solvent for pure O_2_ chemistry [118]. CO_2_ is produced on a very large scale as a chemical waste product and thus is available in large quantities. There are certain features of CO_2_ that make it desirable for use as a solvent, and specifically for reactions utilizing O_2_ [119]. CO_2_ is inert because it is already oxidized and therefore cannot react further with O_2_. CO_2_ has a relatively low critical point (*T*_c_ = 31.1 °C, *P*_c_ = 72.9 bar). ScCO_2_ has interesting physical properties such as zero surface tension and high diffusivity. O_2_ is completely miscible with scCO_2_ thus the reaction will not be driven by mass transfer from the gas to the liquid phase. Using CO_2_ as a solvent is advantageous over conventional solvents as it is miscible with oxygen; therefore, only a small excess, or sometimes even stoichiometric amounts, of oxygen are needed in these reactions. However, product extraction after the reaction can be challenging. Leitner, Greiner and co-workers described a continuous-flow system capable of pilot scale production, although not specifically for a reaction utilizing O_2_ [120]. Leitner and co-workers demonstrated a proof-of-concept Pd-catalyzed aerobic oxidation of primary alcohols to their corresponding aldehydes using scCO_2_ as reaction medium in flow (Scheme 28a) [121]. To circumvent catalyst deactivation, a giant Pd cluster [Pd_561_-phen_60_(OAc)_180_] was stabilized by a poly(ethylene glycol) (PEG)-1000 matrix to circumvent Pd deactivation, and immobilized within a flow reactor. It is important to ensure good mixing between O_2_ and CO_2_ to form a homogeneous mixture before the introduction of the substrate. The catalyst demonstrated reasonable activity and selectivity under unoptimized flow conditions after a single pass.

Poliakoff and co-workers reported the optimization of the oxidation of 2-octanol over a packed bed reactor (5% Pt + 1% Bi on Al_2_O_3_) using scCO_2_ (Scheme 28b) [122]. The system afforded 2-octanone in a consistent 75% yield for 5 h with no evidence for catalyst deactivation and no formation of the octene shown. Subsequently, the system was applied for the oxidation of a number of secondary alcohols (11 examples, 10–75% yield).

**Scheme 29a,b:**
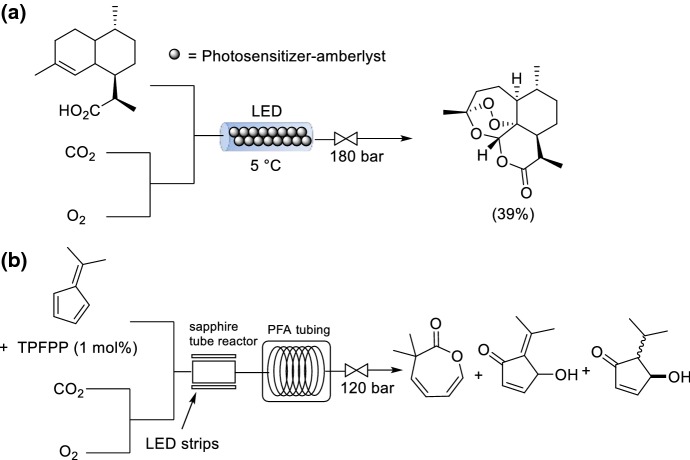
Photooxygenations using liqCO_2_ as (co)solvent. **a** Production of artemisinin. **b** Oxidation of 3-substituted oxepinones

Larger scale processes utilizing ^1^O_2_ normally require non-flammable halogenated solvents to ensure safe operation. ScCO_2_ is a greener alternative to these halogenated solvents. ScCO_2_ has an additional benefit that it lengthens the lifetime of ^1^O_2_ (5.1 ms). An early example by George, Poliakoff and co-workers was the photooxygenation of α-terpinene and of citronellol using immobilized photosensitisers [123]. More recently the same group have examined using liquid CO_2_ (liqCO_2_) for conducting aerobic oxidations [124]. The vapor pressure of liqCO_2_ can be much lower than for scCO_2_, which can enable a reduced pressure limit for the reactor. The production of artemisinin was achieved using liquid CO_2_ as solvent with EtOAc or PhMe as co-solvent over an immobilized dual function solid acid/photocatalyst (Scheme 29a). It can be difficult to achieve a rapid enough reaction rate at the conditions for liquid CO_2_, which may limit its widespread applicability. Very recently, the same group also reported the photooxygenation of a range of fulvenes to 3-substituted oxepinones in liqCO_2_ (Scheme 29b). However, in this case they used a CO_2_-soluble porphyrin photosensitizer [5,10,15,20-tetrakis-(pentafluorophenyl)porphyrin, TPFPP] [125]. The reactive intermediate was generated within a sapphire tube reactor irradiated with LEDs strips, and the intermediate then decomposed under thermal irradiation to yield the 3-substituted oxepinones. The ratio of the different products formed could be controlled by varying the reaction temperature in the two different reactors, co-solvent selection, substrate concentration and CO_2_ flow rate.

### Water

Water is a green and non-flammable solvent, thus, on these terms, water is the ideal solvent for aerobic oxidations. However, a significant limitation associated with using water within flow reactors is that the inherent carbon richness of organic substrates mean that most do not dissolve in water, causing slow reaction rates. Uozumi and co-workers studied the aerobic oxidation of alcohols in H_2_O within a catalyzed by platinum nanoparticles dispersed in an amphiphilic polymer within a continuous flow reactor, but very low substrate concentrations were used (10–100 µM) [126]. The likelihood of multiple phases complicates the development of a flow process due to the multiple phases present. One approach to avoid multiple phases is through the dissolution of organic compounds and O_2_ within a single phase by operating in the supercritical regime for water.

**Scheme 30:**
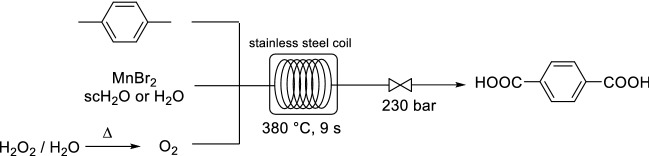
Continuous flow oxidation of para-xylene to terephthalic acid in supercritical H_2_O

As a proof of concept study, Poliakoff and co-workers demonstrated the aerobic oxidation of methylaromatic compounds to their corresponding carboxylic acid derivatives by using manganese(II) bromide as catalyst and scH_2_O as the reaction medium [127]. H_2_O has a high critical point (*T*_c_ = 374 °C, *P*_c_ = 221 bar). Even at high temperatures, below the supercritical regime H_2_O still displays some interesting properties. In a more recent example, the same group studied the selective aerobic oxidation of para-xylene to terephthalic acid at both subcritical and supercritical conditions (Scheme 30) [128]. O_2_ was generated from the high temperature decomposition of H_2_O_2_ in a pre-mixer. Subsequently, the same group reported the identification of improved catalyst systems for the reaction [129]. Nonetheless, the high critical point of H_2_O makes the use of scH_2_O less synthetically relevant for pharmaceutical applications.

## Novel Reactor Developments

The recent developments in reactor technologies for handling O_2_ have aimed to address the challenge associated with the poor solubility of O_2_ in water and organic solvents. Thus, efforts have focused on reducing residence times through the enhancement in the mass transfer from the gas to the liquid phase without the need to employ high pressures, which are often undesired. Recent reactor designs have focused on maximizing the interfacial area between the gas and liquid phase to enhance mass transfer.

**Scheme 31:**
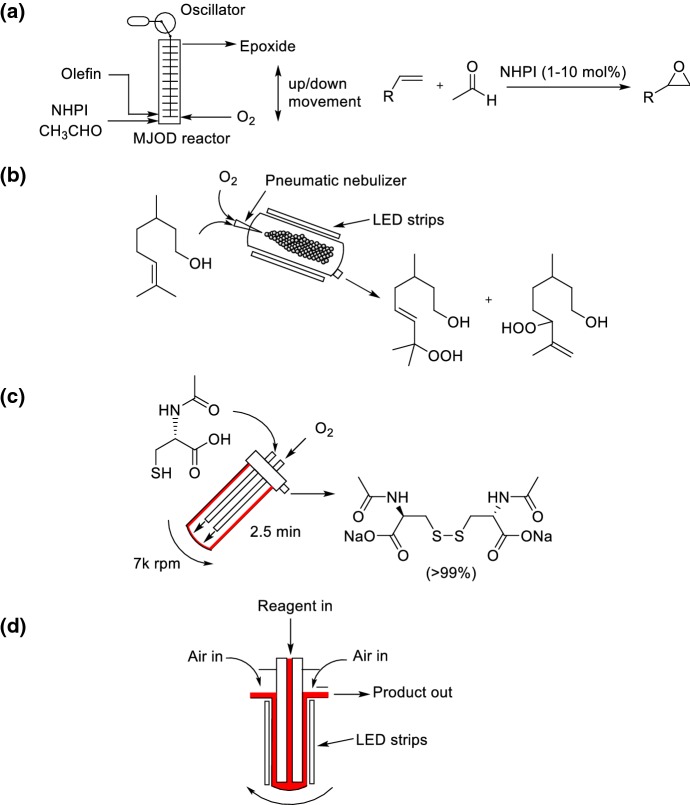
Reactor solutions for handling liquid phase aerobic oxidations. **a** Multi-jet oscillating disk configuration for the Minisci epoxidation of olefins by NHPI. **b** NebPhotOX configuration for the photooxidation of β-citronellol. **c** VFD configuration for the oxidation of *N*-acetyl-l-cysteine. **d** Photochemical vortex reactor

The laboratories at the University of Bergen and at Fluens Synthesis developed a novel reactor platform called the multi-jet oscillating disk (MJOD) reactor [130]. An electric motor powers the up–down movement of a piston by a variable-frequency and variable-amplitude oscillator (Scheme 31a). The reaction mixture moves from one cavity to the next, and is pushed up the reactor through the jets of the MJOD disks. The reactor was applied to the organocatalyzed Minisci aerobic epoxidation of olefins by *N*-hydroxyphthalimide (NHPI) [131]. The batch process for this reaction suffers from the limitation of long reaction times (24–48 h), which limits the efficiency and throughput of the process. The changes in the flow created by the oscillating disks resulted in very good mixing, which accelerated reaction rates and enabled residence times of 1–4 h.

A nebulizer-based continuous-flow reactor has been developed for the photochemical ^1^O_2_ chemistry (NebPhotOX) by Vassilikogiannakis and co-workers at the University of Crete [132]. A solution containing the substrate and the photosensitizer is nebulized by using pure O_2_ or air into a chamber that is enclosed by LED light strips to form ^1^O_2_ as the reactive intermediate. The NebPhotOx system was used for the photooxidation of *β*-citronellol (Scheme 31b). The pneumatic nebulizer generates aerosols consisting of fine droplets with an approximate 60 μm average diameter corresponding to a droplet-specific surface areas of 100,000 m^2^ m^−3^. The same group also reported the synthesis of cyclopent-2-enones from furans using the NebPhotOx reactor in a similar manner [133].

Raston and co-workers have developed a vortex fluidic device (VFD) for accelerating and increasing the efficiencies of organic reactions [134]. The dynamic thin film is generated by continuously adding a fluid from jet feeds to a rapidly rotating surface (Scheme 31c). The reaction mixture is rotated at very high speeds (up to 9000 rpm) to produce the liquid phase as a thin film, thus providing a large surface area between the liquid and gas phases. The reactor system was demonstrated on the aerobic oxidation of thiols to disulfides [135]. In particular, the aerobic oxidation of *N*-acetyl-l-cysteine in water was investigated. Full conversion was achieved in less than 2.5 min residence time within a VFD. The aerobic oxidation within a VFD configuration performed significantly better compared to in batch where only 5% conversion was observed after 1 h reaction time. However, the system was not compared to a segmented flow reactor setup.

George, Poliakoff and co-workers at the University of Nottingham recently reported the construction of a thermal and photochemical “vortex reactor” that uses a rapidly rotating cylinder to generate Taylor vortices (Scheme 31d) [136]. The vortices result in a high interfacial area between the gas and liquid phases, thus enabling rapid dissolution of oxygen into the liquid phase. An interesting feature of the reactor system is that it draws air in from the laboratory so does not specifically need pressurized oxygen from a cylinder, with the optimal uptake of air observed at 4000 rpm. The reactor was demonstrated for a number of reaction systems that utilize ^1^O_2_ as a reagent, including the photooxygenations of α-terpinene and furfuryl alcohol and the photodeborylation of phenyl boronic acid. The system was also applied successfully to develop a single process for a three-step synthesis of artemisinin from artemisinic acid.

**Scheme 32:**
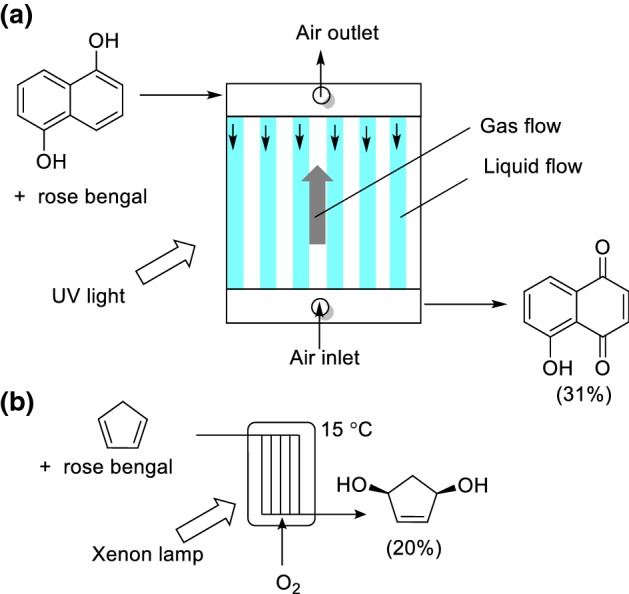
Falling film reactor. O_2_ can flow above the liquid flow either upward or downward (not shown) for the photooxygenation of **a** 1,5-dihydroxynaphthalene and **b** cyclopentadiene

The falling film microreactor was developed by the Institut für Mikrotechnik (IMM, Mainz). The liquid phase flows through microchannels under gravity to form a thin liquid layer, which is as thin as 20 µm in some instances. The gas input then flows co- or counter-currently to the liquid phase with specific phase interfaces of up to 20,000 m^2^m^−3^ generated. Oelgemöller and co-workers studied the photooxygenation of 1,5-dihydroxynaphthalene within a falling film reactor. A 31% yield could be achieved in 160 s residence time (Scheme 32a) [137]. Similarly, Jähnisch and Dingerdissen reported the implementation of a falling film microreactor for the photooxygenation of cyclopentadiene (Scheme 32b) [138]. There was a very small inventory of the endoperoxide produced at any one time, thus improving the inherent safety.

## Conclusion

Liquid phase aerobic oxidation reactions offer a valuable alternative to classical oxidation methods using stoichiometric quantities of toxic inorganic oxidants. The challenges (efficient mixing, safety, catalyst decomposition) associated with the use of O_2_ for organic synthesis can be better addressed through the implementation of continuous flow technology, which can improve reaction reproducibility and provide robust scale-up options. The selection of examples summarized in this review is clear evidence that many aerobic oxidation transformations can be performed effectively and safely under continuous flow conditions. Even pure O_2_, as opposed to synthetic air, can be safely harnessed in particular instances to provide highly convincing synthetic and manufacturing benefits. Nevertheless, the utilization of continuous flow reactors still poses significant challenges in terms of cost and lack of available infrastructure and expertise available within the synthetic chemistry community. We are convinced that, for environmental, economic, regulatory and synthetic reasons, continuous flow aerobic oxidations will be embraced by scientists and engineers within academic laboratories, and the pharmaceutical and fine chemical manufacturing industries, where further exciting developments can be anticipated in the coming years.

## Abbreviations

AcOH: Acetic acid
Bpy: 2,2′-Bipyridine
CrO_3_: Chromium oxide
DMSO: Dimethyl sulfoxide
FEP: Fluorinated ethylene propylene
H_2_: Hydrogen
HNO_3_: Nitric acid
H_2_O_2_: Hydrogen peroxide
HPLC: High performance liquid chromatography
K_2_CO_3_: Potassium carbonate
KHMDS: Potassium bis(trimethylsilyl)amide
KMnO_4_: Potassium permanganate
LED: Light emitting diode
MnO_2_: Manganese oxide
Mt/a: Megatonne per annum
N_2_: Nitrogen
NaHMDS: Sodium bis(trimethylsilyl)amide
NMI: *N*-Methylimidazole
NMP: *N*-Methyl-2-pyrrolidone
O_2_: Oxygen
^1^O_2_: Singlet state oxygen
^3^O_2_: Triplet state oxygen
*P*: Pressure
PFA: Perfluoroalkoxy
PTSA: *p*-Toluenesulfonic acid
*T*: Temperature
TBAI: Tetrabutylammonium iodide
TEMPO: 2,2,6,6-Tetramethylpiperidine *N*-oxyl
TFA: Trifluoroacetic acid
TfOH: Trifluoromethanesulfonic acid
TMEDA: Tetramethylethylenediamine
